# Affective meaning from unlabeled biological motion: Associations with social–perceptual sensitivity

**DOI:** 10.1371/journal.pone.0352873

**Published:** 2026-09-01

**Authors:** Mark Jaime, Elizabeth B. daSilva

**Affiliations:** Indiana University, Columbus, Indiana, United States of America; Northeastern Illinois University, UNITED STATES OF AMERICA

## Abstract

Although the thoughts, feelings, and intentions of others are not directly accessible to perception, they are often inferred from observable behavior. Human movement may be especially important in this process because it provides continuously unfolding spatiotemporal structure through which socially meaningful interpretations can emerge. Prior work has shown that observers can derive social and affective meaning from movement, even when it is reduced to point-light displays (PLDs). Less is known, however, about whether perceivers can form affective judgments from movement when contextual and categorical emotion cues are reduced while natural individual variation in expressive movement is preserved, and whether this ability is related to social–perceptual sensitivity. In the present study, participants viewed unlabeled PLDs depicting bodily movements originally generated by actors attempting to express anger, fear, or happiness. Participants were not informed of the intended affective state of each PLD and provided valence–arousal ratings based only on the movement patterns they observed. Ratings were transformed into angular representations within circumplex affective space and compared with theoretically derived reference locations associated with the actor-instructed movement conditions. Results showed that higher social–perceptual sensitivity was associated with valence–arousal judgments positioned closer to the theoretically derived circumplex reference locations associated with the movement conditions. This relationship differed across movement conditions, with the strongest associations observed for anger-related PLDs relative to happiness-related PLDs. Together, these findings suggest that social–perceptual functioning contributes to how perceivers derive affective meaning from human movement when contextual and categorical emotion cues are reduced. More broadly, the findings support the view that affective meaning derived from movement reflects an interaction between the spatiotemporal organization of movement and the perceiver’s attunement to that organization.

## Introduction

Social understanding depends in part on perceiving how other bodies move. Although affective states and intentions are not directly observable as private experiences, they become socially available through patterned changes in movement [1]. Human movement is especially important because it unfolds continuously and exhibits structured spatiotemporal and kinematic organization, providing information not only about what actions are being performed, but how they are carried out. For example, differences in acceleration, expansiveness, contraction, and rhythm can alter how otherwise similar actions are perceived, shaping whether movement appears tense, relaxed, or threatening [2–4]. Consistent with this perspective, research on nonverbal expression suggests that movement itself may provide structured perceptual information through which affective inference may be supported [5–7]. These observations have motivated growing interest in how dynamic movement relates to social and affective inference.

Point-light displays (PLDs) provide a powerful method for examining these questions because they preserve the dynamic organization of biological motion while minimizing many other sources of social information. By reducing the visible body to moving points, PLDs remove surface form, texture, facial detail, and other contextual information while retaining the spatiotemporal structure of movement itself [8]. Despite this reduction, observers reliably perceive human action from such displays, and biological motion has been shown to support judgments about action identity, affective qualities, person characteristics, and aspects of intention [3,9–11]. The ability to derive affective meaning from movement is not limited to human biological motion. In the classic Heider–Simmel animations, observers readily attribute goals, intentions, and emotional states to simple geometric shapes based largely on their movement patterns [12]. These findings suggest that socially meaningful interpretations can be derived from dynamic movement patterns even when many conventional social cues are absent.

The relationship between movement and social inference is evident even early in development. Infants show sensitivity to biological motion, including the ability to discriminate coherent from scrambled point-light movement and expectations that such movement should appear upright [13,14]. With increasing experience, infants also detect violations in familiar movement structure and object dynamics [15–17] and begin to anticipate aspects of goal-directed action and attentional orientation [18]. Together, these findings suggest that perceptual experience with structured movement may contribute to the emergence of increasingly sophisticated interpretations of action. These developmental accounts raise the possibility that sensitivity to dynamic movement becomes functionally linked to social perception, including how perceivers derive affective meaning from bodily motion [9,17,19,20].

Autism spectrum disorder (ASD) provides another important context for linking movement perception with social understanding. ASD is centrally characterized by differences in social communication and social cognition, including differences in mental-state attribution and broader social–cognitive interpretation [21,22]. If social understanding and movement perception were fully independent domains, there would be little reason to expect social-cognitive differences in ASD to be accompanied by differences in how movement is interpreted. However, several lines of work suggest that movement-based interpretation is also altered in ASD. Action observation, imitation, and mirror-system accounts have linked autism to differences in perceiving and mapping others’ actions [23,24]. More directly relevant to the present study, research using PLDs and Heider–Simmel-type displays suggests that autistic individuals may differ in how socially meaningful interpretations are derived when movement is presented with limited contextual information. For example, individuals with ASD frequently produce fewer social attributions to moving geometric shapes than neurotypical observers [25–27], and a recent systematic review concluded that autistic individuals tend to show greater difficulty on biological motion tasks that place higher demands on social perceptual and cognitive interpretation [28]. Taken together, this literature suggests that movement perception and social understanding are not easily separable: differences in ASD appear not only in social-cognitive interpretation broadly, but also in how dynamic movement is mapped onto socially meaningful interpretations [29–31].

An important question raised by this literature is what kinds of affective meaning perceivers can derive from human movement when movement is the primary available cue. If socially meaningful interpretations can be formed from reduced movement displays, then a related question is whether such interpretations vary with broader social–perceptual functioning. Despite the relevance of this issue for understanding how perceivers derive social meaning from biological motion, relatively little work has examined whether affective judgments derived from movement alone are related to broader social–perceptual functioning. For example, Alaerts et al. [32], in a study primarily focused on gender differences in action and emotion recognition from point-light displays, reported that performance in judging whether point-light movements appeared happier, sadder, or angrier than a neutral prime was positively associated with scores on the Reading the Mind in the Eyes Test (RMET; [33]. However, the significance of this relationship was interpreted largely within the context of generalized emotion-recognition abilities across facial and bodily expression domains. RMET performance and biological-motion judgments were implicitly treated as related forms of socio-emotional recognition differing mainly in whether socially relevant information was conveyed through facial or bodily cues.

The present conceptual framework approaches this relationship somewhat differently. Historically, the RMET was not originally developed as a facial emotion-recognition task. Rather, it was designed to assess the ability to infer subtle and often complex mental states from highly restricted static visual information confined to the eye region of the face. Many RMET items involve nuanced social-cognitive states extending beyond basic emotional expressions alone. Thus, the relationship between RMET performance and biological-motion judgments likely reflects more than a generalized ability to recognize emotion from facial and bodily expressions. Moreover, RMET performance and biological-motion judgments rely on fundamentally different forms of perceptual information: the RMET requires inference from static visual cues, whereas biological-motion tasks require interpretation of dynamically unfolding movement patterns. From this perspective, the central issue is not whether observers correctly recognize discrete emotional categories from biological motion, but whether affective meaning can be derived from movement alone and whether this process is related to broader social–perceptual functioning.

To examine this question, participants in this study viewed PLDs depicting emotionally expressive movements and generated valence and arousal judgments based solely on the movement patterns they observed. We emphasize that, although the point-light displays were originally generated from actors instructed to express particular affective states, participants viewing the displays were never told which emotion the actor was attempting to convey. Participants did not choose among predefined emotion labels such as anger, fear, or happiness, nor were they asked to decide whether a movement was “correctly” expressing a particular emotional category. Instead, participants simply viewed unlabeled PLDs and generated valence and arousal judgments based solely on the movement patterns they observed. We hypothesized that higher RMET scores would be associated with valence–arousal judgments that were more systematically positioned relative to theoretically derived circumplex reference locations associated with the actor-instructed movement condition [34,35].

In addition to associations with RMET performance, we also examined associations with scores on the Autism-Spectrum Quotient (AQ; [36]) as an exploratory individual-difference measure. Unlike the RMET, the AQ is not a performance-based measure of social–perceptual inference, nor is it a diagnostic assessment of autism spectrum disorder. Rather, it provides a self-report index of broader autism phenotype traits in the general population. Importantly, studies looking at autistic-like trait variation measured in neurotypical populations should not be treated as interchangeable with studies with clinically verified ASD samples, although both literatures have examined biological motion and social perception. Nonetheless, because differences in social cognition are a central feature of autism and prior work has linked autism-spectrum traits to differences in the perception of emotion from bodily movement [37], it was plausible that broader autism-related trait variation might also relate to affective judgments derived from biological motion. Accordingly, AQ scores were examined to determine whether valence–arousal judgments from biological motion were related specifically to RMET-indexed social–perceptual functioning, to broader autism-related trait variation within a neurotypical sample, or to both.

We also expected that the relationship between social–perceptual functioning and affective interpretation would differ across affective movement conditions. Prior work demonstrates that affective states can be expressed and interpreted through bodily movement, including point-light displays [3,9,19]. However, affective movement conditions may differ in how consistently they are interpreted under reduced movement-viewing conditions. Related work with whole-body expressions further suggests that happiness may show greater variability or reduced discriminability relative to several other affective states [38]. Accordingly, we tentatively expected the relationship between social–perceptual functioning and affective interpretation to vary across movement conditions, with stronger associations for anger- and fear-related movements than for happiness-related movements.

## Materials and methods

### Participants

A total of 140 participants took part in the study. Participants were recruited from Indiana University Columbus undergraduate students through course announcements, the psychology majors listserv, campus flyers, and email invitations. Additional community participants were recruited through word of mouth from the research team. Recruitment and consent materials described the study as examining how human body movements may reveal what a person is thinking or feeling, as well as broader person-level characteristics. Eligible participants for the present analyses were adults 18 years of age or older who provided informed consent. No exclusion criteria other than age were applied. Participant demographics, including age and sex distributions are reported in Table 1. Additional demographic variables were not collected. All participants received a small monetary gift (e-card) for participation. Study procedures were approved by the Indiana University Institutional Review Board (Protocols 1803460554 and 12195) and all procedures in this study were conducted in accordance with institutional guidelines. Recruitment took place between January 18, 2021 and November 16, 2021. All participants provided a written informed consent prior to participation. Participation was voluntary, and no minors were included in the study.

**Table 1:** Demographic characteristics of the participant sample.

Characteristic	Main Sample (N = 140)
**Age (years)**	*M* = 25.94, *SD* = 8.82
**Sex**	
Male	35 (25.0%)
Female	104 (74.3%)
No answer	1 (0.7%)

### Stimuli

Fifteen point-light displays (PLDs) used in the present study were selected from a larger stimulus set developed in Lewis et al. [39], where detailed procedures for movement recording and PLD generation are described. PLDs are visual representations of human movement in which the body is reduced to a small number of moving points positioned at major joints and body regions. In these displays, observers do not see facial features, body shape, clothing, texture, or other surface characteristics typically available in ordinary visual perception. Instead, PLDs preserve only the movement patterns generated by the moving body itself [8]. As described in Lewis et al. [39], participants were recorded while attempting to express anger, fear, and happiness through full-body movement. Movements were captured using a Microsoft Kinect camera and the Kinect-based Biological Motion Capture (KBC) toolbox [40], which extracted the spatial positions of major body points across time. These recordings were then converted into PLDs.

For the present study, PLDs from 3 affective movement conditions (anger, fear, happiness) were selected from 15 different actors, yielding a total of 45 unique movement stimuli (15 anger, 15 fear, 15 happiness). The mean duration of the PLDs was 6.24 seconds (SD = 3.66). Anger displays tended to be longer in duration (M = 8.00 s, SD = 5.26) than fear displays (M = 5.87 s, SD = 2.70) and happiness displays (M = 4.87 s, SD = 1.25). A repeated-measures ANOVA revealed a significant effect of affective movement condition on PLD duration, F(2, 28) = 5.40, p = .020 (Greenhouse–Geisser corrected), although Bonferroni-adjusted pairwise comparisons were not statistically significant (anger vs. fear: p = .16; anger vs. happiness: p = .07; fear vs. happiness: p = .29). These duration differences were not experimentally constrained because the original stimulus recordings were developed in Lewis et al. [39] to preserve relatively natural variability in expressive movement style across actors and affective movement conditions rather than impose highly standardized portrayals of emotion. In that study, the primary focus concerned naturally varying movement kinematics. Accordingly, variability in movement duration was retained as part of the expressive movement structure of the displays used in the present study.

A separate sample of independent raters (N = 40) was used to evaluate whether the PLDs elicited affective impressions in observers rather than appearing as arbitrary movement patterns. The goal of this validation was to determine whether movements generated from the 15 PLD actors instructed to express anger, fear, and happiness through bodily movement would produce corresponding affective impressions in naïve observers who were not aware of the affective state the actor had been instructed to express. Thus, the 40 raters did not know whether a given display had been generated from an actor attempting to express anger, fear, or happiness.

For each point-light display, the 40 independent raters answered three separate questions: “How angry are the movements?”, “How fearful are the movements?”, and “How happy are the movements?” Responses were made using 7-point Likert scales ranging from 1 (“not at all”) to 7 (“very much”). Thus, each display received separate anger, fear, and happiness ratings rather than being assigned to a single emotional category. Trials were presented in pseudo-randomized order in blocks by actor, with the presentation order of actors randomized across participants. Stimulus ratings were collected online using the Qualtrics survey platform (Qualtrics, Provo, UT).

Logistic regression analyses were then used to examine whether displays generated from actors instructed to express anger tended to receive higher anger ratings, whether displays generated from actors instructed to express fear tended to receive higher fear ratings, and whether displays generated from actors instructed to express happiness tended to receive higher happiness ratings. The analyses indicated that higher anger ratings were associated with an increased likelihood that a display had been generated from actors instructed to express anger through bodily movement, β = 0.178, SE = 0.026, z = 6.82, p < .001, OR = 1.20, 95% CI [1.14, 1.26], Δ deviance = 46.8, χ²(1) = 46.8, p < .001. Similarly, higher fear ratings were associated with displays generated from actors instructed to express fear, β = 0.241, SE = 0.027, z = 8.93, p < .001, OR = 1.27, 95% CI [1.21, 1.34], Δ deviance = 81.5, χ²(1) = 81.5, p < .001, and higher happiness ratings were associated with displays generated from actors instructed to express happiness, β = 0.154, SE = 0.030, z = 5.16, p < .001, OR = 1.17, 95% CI [1.10, 1.24], Δ deviance = 26.5, χ²(1) = 26.5, p < .001. At the same time, substantial overlap remained across rating distributions, indicating that observers’ affective impressions of the displays were graded rather than strongly categorical under reduced point-light conditions. These relationships showed that the point-light displays retained emotionally meaningful information and therefore provided a suitable stimulus set for examining participants’ valence and arousal judgments of affective movement.

### Procedure

Participants viewed all 45 point-light displays and rated each PLD on two affective dimensions: valence and arousal using separate 7-point Likert scales. For valence, participants were asked, “To what extent does the movement appear negative or positive?” with response options ranging from “Very Negative” to “Very Positive.” For arousal, participants rated the extent to which “the movement” appeared “Very Relaxed” to “Very Excited.” Importantly, the rating prompts explicitly referred to “the movement” rather than to the actor or a predefined emotional category, thereby directing participants’ judgments toward the affective qualities conveyed through the movement patterns themselves. Participants were never told which emotion the actor had originally been instructed to express when the movement was recorded. Thus, participants did not know whether a given PLD had been generated from an actor attempting to express anger, fear, or happiness through bodily movement. Instead, judgments were based entirely on the movement patterns conveyed by the PLDs themselves.

All ratings were administered and recorded using the Qualtrics online survey platform (Qualtrics, Provo, UT). The study was completed remotely through Qualtrics rather than in a supervised laboratory setting. On each trial, participants viewed a PLD video embedded within the survey interface and were instructed to press play before answering the rating questions. The video remained available on the page, allowing participants to replay the PLD if needed before submitting their responses. Allowing replay reduced the likelihood that a participant’s response would reflect a missed or interrupted viewing rather than their judgment of the display. Rating questions appeared directly beneath the video.

### Angular distance transformation

To quantify how participants mapped each point-light display into affective space, valence and arousal ratings were transformed into angular positions within the Circumplex Model of Emotion [34,35]. This transformation did not treat participant responses as categorical emotion-recognition judgments. Rather, the approach quantified the directional positioning of participants’ valence–arousal judgments within a two-dimensional affective space. Raw valence and arousal ratings were first recentered so that the neutral midpoint of each 7-point scale (4) corresponded to the origin (0,0). Valence was represented on the horizontal axis and arousal on the vertical axis. Angular positions were then calculated using the arctangent function:


$$\begin{array}{c} \theta =\text{atan2}(Arousal_{c},\;Valence_{c}) \end{array}$$

where $Arousal_{c}$ and $Valence_{c}$ represent the recentered arousal and valence ratings, respectively. Thus, each participant’s valence–arousal judgment was represented as a directional angle within circumplex affective space rather than as a discrete emotion category judgment.

**Fig 1:**
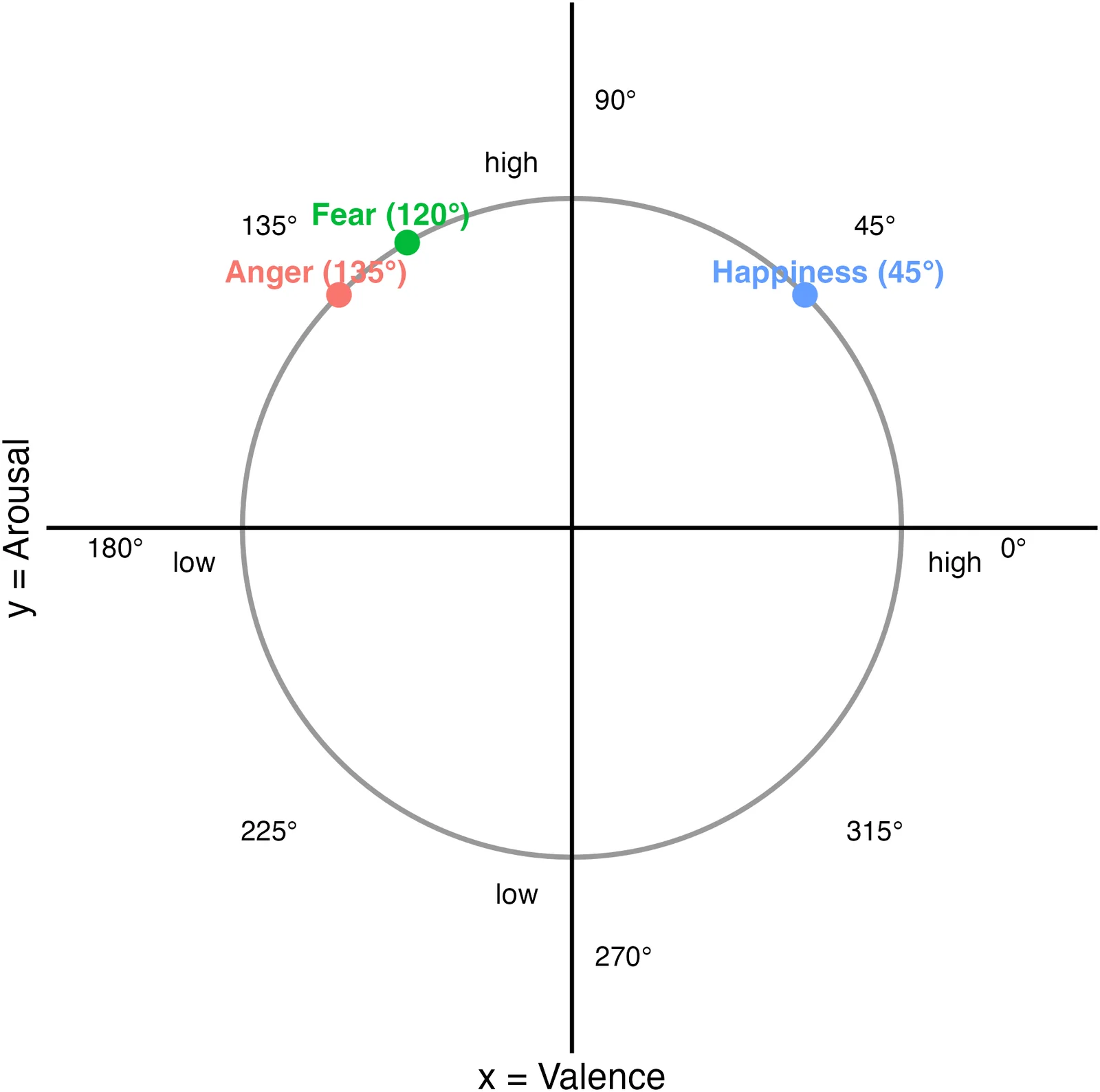
Schematic representation of the circumplex model showing the theoretical reference positions of Anger (135°), Fear (120°), and Happiness (45°) within valence–arousal space. The figure illustrates the theoretically derived reference angles used in the angular distance analysis and does not represent participant ratings.

We next specified theoretical reference angles for the three stimulus conditions using canonical depictions of affective organization reported in circumplex models of emotion [34,35,41,42]. Within the circumplex framework, happiness is generally represented as positively valenced and relatively high in arousal, placing it in the upper-right region of affective space. Fear and anger, in contrast, are both typically represented as negatively valenced and relatively high in arousal, placing them in the upper-left region of the circumplex. Based on prior circumplex depictions reported in the literature, we approximated angular reference positions of Happiness ≈ 45°, Fear ≈ 120°, and Anger ≈ 135° for use in the present analyses (Fig 1). These values should not be interpreted as objectively “correct” emotional coordinates for each movement display. Rather, they served as theoretically motivated reference locations derived from prior circumplex models of affect [34,35].

Angular distance was calculated as the smallest circular difference between a participant’s observed rating angle and the corresponding theoretical reference angle for a given movement condition. Because the analysis operated within circular affective space, angular differences wrapped around the circumplex rather than increasing indefinitely in a linear manner. Thus, angular-distance values always reflected the shortest directional separation between two locations within circumplex space. For example, if the theoretical reference angle for a movement condition was 135°, participant judgments of 125° and 145° would both produce an angular-distance value of 10°, because both judgments deviated from the reference location by the same amount despite falling on opposite sides of the reference angle. Angular-distance values therefore reflected the relative position between a participant’s valence–arousal judgment and the theoretical reference location associated with a movement condition. Thus, lower angular-distance values indicate judgments positioned closer to the theoretical reference location within circumplex space, whereas higher values indicate judgments positioned farther away from that location. Accordingly, angular distance served as a relational index describing how participants’ valence–arousal judgments were positioned relative to theoretically derived circumplex reference locations, rather than as a measure of categorical emotion-recognition accuracy.

### Individual difference measures

Participants completed the Reading the Mind in the Eyes Test (RMET; [33]), a performance-based measure in which participants selected, from four alternatives, the mental-state descriptor that best matched a static image containing only the eye region of the face. The RMET consists of 36 items assessing the attribution of complex mental states from highly restricted facial information. RMET scores served as the primary individual-difference measure in the present study. Participants also completed the Autism-Spectrum Quotient (AQ; [36]), a 50-item self-report measure designed to assess broader autism phenotype traits in adults of normal intelligence. The AQ includes items spanning domains related to social skill, communication, attention switching, imagination, and attention to detail. AQ scores served as a secondary individual-difference measure. The AQ is not an ASD diagnosis.

## Results

### Data completeness

Three participants did not contribute usable valence–arousal rating data and were therefore excluded from the analytic dataset, resulting in a final analytic sample of 137 participants. Across the 45 displays and 137 participants, a total of 6,165 observations were expected. Of these, 5,940 observations were available for analysis, reflecting some skipped trials in which participants did not provide a response. Missing responses were distributed equivalently across anger, fear, and happiness conditions, χ²(2) = 0.00, p = 1.00. Exploratory checks at the individual display level indicated only modest variability in missingness, with the highest stimulus-level missingness proportion equal to 6.57% (9 missing responses out of 137 expected observations). Participant-level missingness was limited, with participants missing an average of 1.64 out of 45 expected ratings. The highest level of participant-specific missingness involved 39 missing trials for a single participant. Because analyses were conducted using mixed-effects models, participants with incomplete data were retained rather than excluded entirely. Thus, participants who completed fewer than all 45 ratings still contributed their available observations to the estimation of the fixed and random effects in the model.

### Descriptive valence–arousal rating patterns

**Fig 2:**
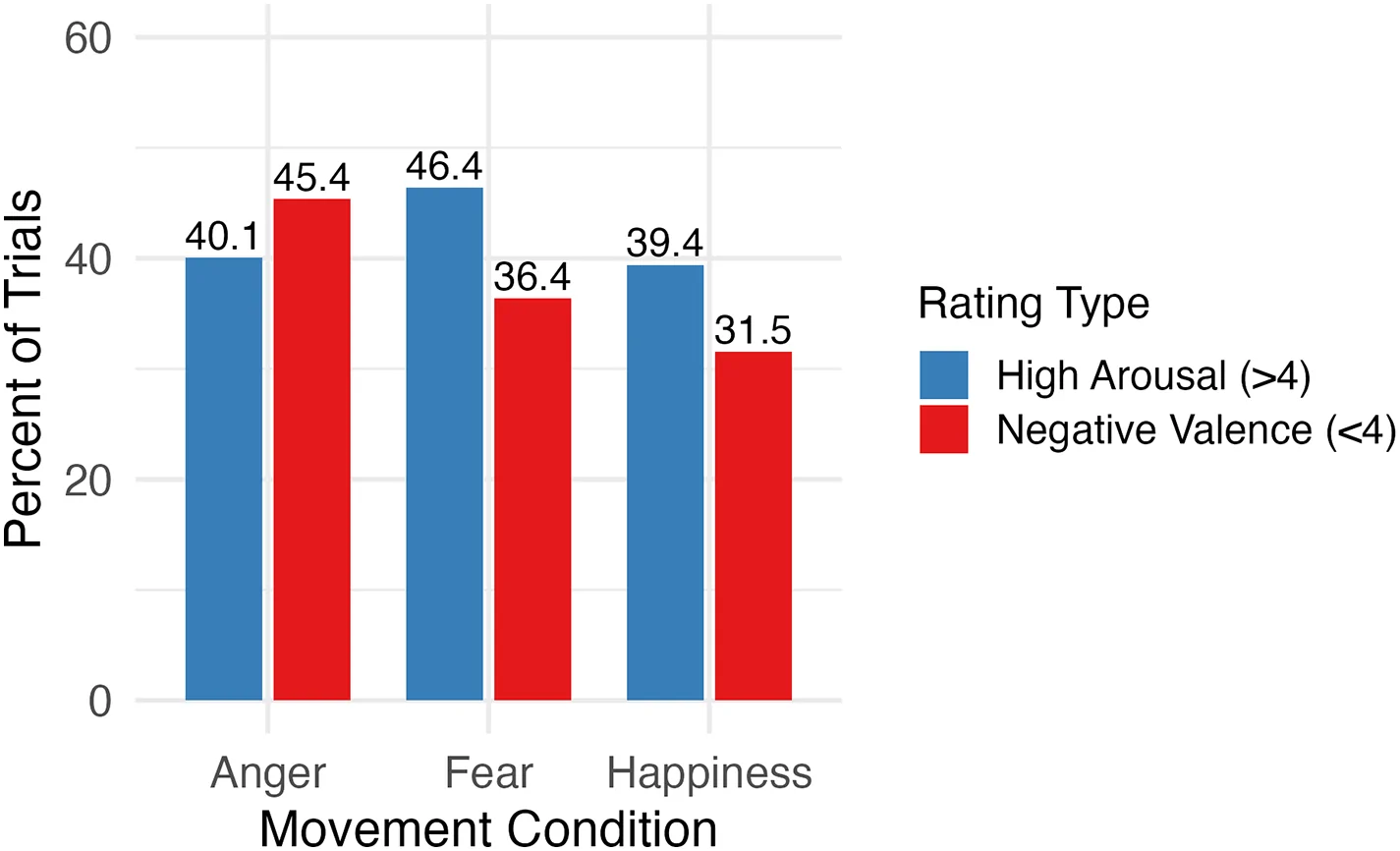
Proportion of negative-valence and high-arousal ratings across actor-instructed movement conditions. Participants were not informed of the actor-instructed affective condition of each PLD. Bars show the proportion of trials rated as negative in valence (< 4; red bars) and high in arousal (> 4; blue bars) for anger, fear, and happiness PLDs.

Before conducting the angular-distance analyses, we examined how participants distributed valence and arousal judgments across the three movement conditions (Fig 2). These descriptive analyses were intended to characterize the overall patterning of participants’ valence–arousal judgments prior to the angular-distance transformation. For valence judgments, anger PLDs most frequently received ratings below the neutral midpoint of the scale (45.4% of trials < 4), compared to fear PLDs (36.4%) and happiness PLDs (31.5%). For arousal judgments, fear PLDs most frequently received ratings above the neutral midpoint of the scale (46.4% of trials > 4), followed by anger PLDs (40.1%) and happiness PLDs (39.4%). Chi-square analyses confirmed that the distribution of negative-valence ratings differed significantly across movement conditions, χ²(2) = 83.09, p < .001, as did the proportion of high-arousal ratings, χ²(2) = 24.09, p < .001.

### Angular distance analyses

To examine whether individual differences in social–perceptual functioning were associated with participants’ positioning of valence–arousal judgments relative to theoretically derived circumplex reference locations, we fit a linear mixed-effects model with angular distance as the dependent variable. Predictors included RMET scores (continuous), Emotion condition (Anger, Fear, Happiness), participant sex, and the RMET × Emotion interaction. A random intercept was included for participant.

### Main effects

The model revealed a significant main effect of RMET, β = –0.85, SE = 0.32, 95% CI [–1.48, –0.23], t(467.50) = –2.69, p = .007, indicating that higher RMET scores were associated with smaller angular-distance values overall. Thus, participants with higher RMET scores tended to position valence–arousal judgments closer to the theoretically derived reference locations associated with the movement conditions.

**Fig 3:**
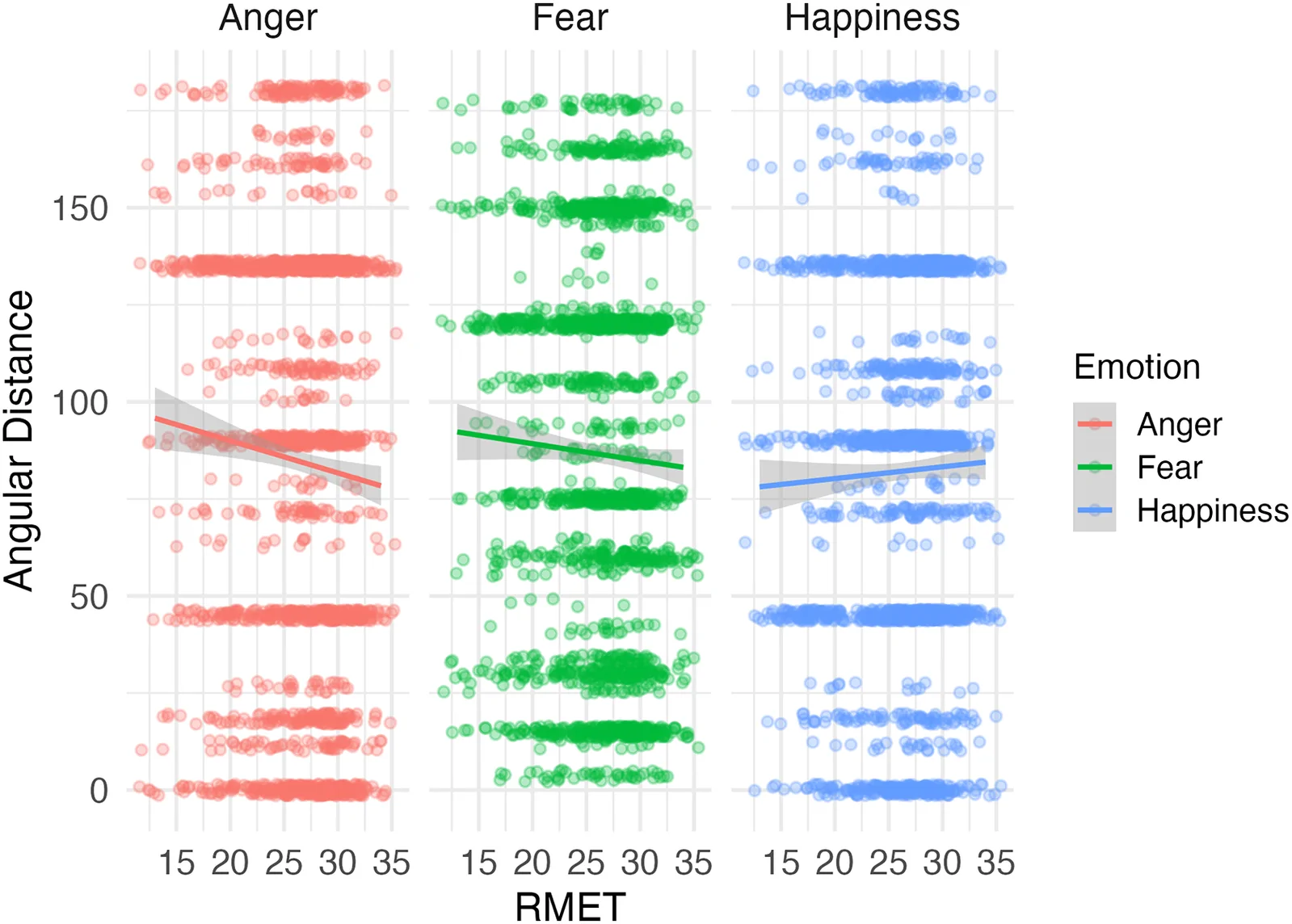
RMET associations with angular distance across movement condition. Angular distance relative to theoretical circumplex reference locations is plotted as a function of RMET (Reading the Mind in the Eyes Test) scores for anger, fear, and happiness point-light displays (PLDs). Points represent trial-level ratings (jittered for visibility), and lines show mixed-effects model predictions with 95% confidence intervals.

There was also a significant main effect of Emotion condition, reflecting mean angular-distance differences across movement conditions; pairwise contrasts are reported below. Including participant sex in the model did not reveal a significant main effect of sex, β = 0.68, SE = 19.64, t(438.20) = 0.04, p = .973, nor did sex alter the primary RMET × Emotion findings.

### RMET × Emotion interaction

The RMET × Emotion interaction was significant, indicating that the relationship between RMET performance and angular distance differed across movement conditions (see Fig 3).

### Post hoc analyses

Pairwise contrasts were conducted to clarify the significant Emotion-condition effect. For mean angular-distance values, fear PLDs showed significantly larger angular-distance values than happiness PLDs, estimate = 4.36, SE = 1.67, 95% CI [0.45, 8.28], z = 2.61, p = .025. Mean angular-distance differences between anger and fear PLDs (p = .56), and between anger and happiness PLDs (p = .25), were not statistically significant.

Pairwise comparisons of RMET slopes across movement conditions indicated that the association between RMET scores and angular distance was significantly stronger for anger PLDs than for happiness PLDs, estimate = –1.13, SE = 0.38, 95% CI [–2.02, –0.25], z = –3.00, p = .008. Slope differences between anger and fear PLDs (p = .55), and between fear and happiness PLDs (p = .12), were not statistically significant. Exploratory analyses using Autism Spectrum Quotient (AQ) scores did not reveal significant associations between AQ and angular distance, nor did AQ reproduce the RMET-related associations observed in the primary analyses.

## Discussion

The present study examined how perceivers form affective judgments from unlabeled point-light displays of human movement when contextual and categorical emotion cues are reduced. Participants were never informed of the actor’s intended affective state and instead generated valence and arousal ratings from movement alone. Participants’ valence and arousal ratings were examined within a circumplex framework of affect, meaning they were represented as angular positions within a two-dimensional valence–arousal space. Based on prior circumplex models [34,35], theoretical reference angles were assigned to the anger, fear, and happiness conditions. Within this framework, angular-distance values indexed how participants’ valence–arousal judgments were positioned relative to the theoretical reference location associated with each movement condition. To examine whether social–perceptual functioning was related to this process, we used performance on the Reading the Mind in the Eyes Test (RMET; [33]) as an index of social–perceptual sensitivity. The RMET requires perceivers to infer subtle mental-state information from highly restricted static cues confined to the eye region of the face. To our knowledge, relatively little prior work has examined how social–perceptual functioning relates to valence–arousal judgments derived from unlabeled point-light movement alone.

In addition, because the central question of the present study was whether affective meaning can be derived from movement alone, it was necessary to establish that the unlabeled PLDs used in the study elicited systematic affective impressions rather than nonspecific impressions of point-light motion. Importantly, analyses conducted on an independent sample indicated that movements generated from different actor-instructed affective states were associated with corresponding affective judgments among observers. Thus, although participants in the present study were never informed of the intended affective states associated with the PLDs, the stimuli themselves had been vetted for their capacity to elicit affective impressions rather than random biological motion.

Our results suggest that perceivers can form systematic affective judgments from movement alone, and that social–perceptual functioning is related to how those judgments are positioned within affective space. Participants with greater social–perceptual sensitivity, as measured by the RMET, tended to position valence–arousal judgments closer to the theoretical reference locations associated with the actor-instructed movement conditions. These findings raise the possibility that socially meaningful affective judgments emerge through the relation between structured movement information and perceiver-level social–perceptual sensitivity. At the same time, the present study did not directly isolate the specific kinematic features contributing to these judgments, and the correlational nature of the design precludes strong causal conclusions regarding the mechanisms underlying these associations. However, questions concerning how socially meaningful information may be organized within movement are difficult to address mechanistically at the outset. Establishing whether systematic relationships exist therefore represents an important first step toward understanding how movement features and perceptual processes may contribute to affective judgments derived from movement alone.

The present findings also suggest a different interpretation of the relationship between RMET performance and biological-motion judgments than has been emphasized in prior work. Alaerts et al. [32], for example, reported associations between RMET scores and point-light emotion judgments within a framework emphasizing generalized emotion-recognition abilities across facial and bodily expression domains. We interpret the present findings somewhat differently. The RMET was not originally developed as a simple facial emotion-recognition task, but rather as a task requiring perceivers to derive socially relevant mental-state information from subtle and highly restricted visual cues confined to the eye region of the face [33]. In contrast, the unlabeled PLDs used in the present study required perceivers to make affective inferences based solely on dynamically unfolding point-light movement. The present findings are therefore not easily characterized as evidence that participants were merely recognizing the same emotional categories across facial and bodily stimulus formats. Rather, the findings suggest that social–perceptual sensitivity may play a role in how perceivers detect and use subtle socially meaningful movement patterns when forming affective inferences from motion alone.

The interaction findings suggest that the relationship between RMET performance and affective judgments varied across actor-instructed affective movement conditions. Specifically, the relationship between RMET performance and angular-distance values was strongest for PLDs generated from actor-instructed anger movements and weakest for PLDs generated from actor-instructed happiness movements, the fear-related pattern was intermediate and less clearly differentiated. Importantly, the interaction findings should be interpreted separately from the overall mean angular-distance differences observed across movement conditions. Happiness-related PLDs produced smaller angular-distance values overall, indicating that participants’ valence–arousal judgments tended to fall relatively closer to the theoretical happiness reference location on average. At the same time, RMET performance showed little association with variation in those happiness-related judgments. In contrast, anger-related movements showed greater overall variability in angular-distance values, but the relationship between RMET performance and those judgments was substantially stronger. Thus, the present findings do not suggest that happiness-related movement is less socially meaningful or less dependent on social–perceptual processing. Rather, the pattern suggests that the relationship between observer-level social–perceptual sensitivity and affective judgments varied across these movement conditions. One possible explanation is that happiness-related movements elicited more consistent affective impressions across observers under the present viewing conditions, thereby leaving less observer-level variability for RMET performance to explain.

Prior work has shown that affective judgments derived from bodily movement and point-light displays can vary across expressive conditions, likely because different forms of affective movement are associated with different patterns of spatiotemporal and kinematic organization [3,9,43]. For example, Atkinson et al. [9] reported that recognition accuracy for dynamic bodily expressions differed across emotions and that exaggerated movement enhanced recognition for some affective states in point-light displays, whereas sadness appeared comparatively less influenced by this manipulation. Similarly, Roether et al. [43] demonstrated that emotional gait perception depends on coordinated variations in posture, movement amplitude, velocity, and spatiotemporal organization, with anger- and happiness-related gait expressions characterized by larger and more activated movement patterns than fear- or sadness-related gait expressions. Other work further suggests that anger-related bodily movement may remain comparatively robust under perceptually reduced viewing conditions. Visch et al. [44], for instance, found that anger perception from bodily expressions remained relatively resistant to image degradation and body segmentation, while Chouchourelou et al. [45] reported enhanced visual sensitivity to angry walkers in point-light displays, potentially related to differences in movement velocity structure.

Likewise, Ikeda and Watanabe [46] observed that explicit detection of coherent biological motion was associated with anger detection but not happiness detection. These findings raise the possibility that the stronger relationship observed for anger-related movements in the present study may reflect differences in the extent to which coordinated movement organization associated with distinct valence–arousal characteristics remained perceptually available across movement conditions. Findings involving fear-related movements were less clearly differentiated than the anger-related findings. One possibility is that fear-related affective information may have been conveyed less consistently through movement alone under reduced point-light viewing conditions, although the present design does not permit strong conclusions regarding the specific movement features contributing to these effects. Future work may benefit from directly quantifying emotion-specific movement features, as prior work suggests that distinct motor components contribute to recognition of different emotions from movement [47].

An additional possibility is that different affective movement conditions placed different interpretive demands on perceivers under reduced point-light conditions. The observed interaction effects may not solely reflect differences in the movement patterns themselves, but also differences in the extent to which perceivers were required to rely on subtle social–perceptual processes when forming affective judgments from perceptually constrained movement information. Prior work has suggested that biological motion perception under reduced viewing conditions depends substantially on observer-level processes related to perceptual organization and social interpretation [10,48]. In addition, Pollick et al. [49] emphasized that biological motion perception involves substantial observer-level variability related to perceptual experience and social-cognitive functioning. This interpretation is also consistent with work suggesting that observers do not rely uniformly on the same movement information across affective conditions. Bachmann et al. [50], for example, reported that perceivers appeared to use different movement information depending on the emotional state being judged, indicating that affective inference from bodily movement may involve different perceptual weighting strategies across expressive conditions. This literature raises the possibility that the stronger relationship observed between RMET performance and angular-distance values for unlabeled PLDs depicting movements generated from anger-related actor instructions in the present study may reflect situations in which affective inference depended more heavily on observer sensitivity to subtle socially relevant movement patterns. However, because the present study did not directly quantify the movement properties contributing to these effects or directly manipulate perceptual ambiguity or observer uncertainty, these interpretations remain tentative.

The present findings are consistent with the view that socially relevant affective meaning may emerge from the coordinated spatiotemporal organization of human movement and that perceivers differ in how effectively they detect and use that information when forming affective judgments. Research on biological motion perception has long demonstrated that observers can derive socially relevant information from highly reduced movement displays, including intentions, affective states, and interpersonal interactions, even when structural visual information is substantially limited [8,10,48,51]. Runeson and Frykholm [11] argued that human movement is systematically shaped by the biomechanics and temporal coordination of the body, thereby permitting differences in affective state or social intention to be reflected in observable differences in movement timing, posture, trajectory, and coordination across the body. Later work demonstrated that subtle variations in movement kinematics can systematically influence the perception of social intentions, cooperation, competition, and affective states. For example, Becchio et al. [52] reported that cooperative and competitive reach-to-grasp actions differed in movement velocity, trajectory height, and temporal coordination, while Georgiou et al. [53] demonstrated that subtle variations in body movement kinematics can systematically influence social judgments about intention and affective state. Prior research also suggests that observers differ in how effectively they use socially relevant movement patterns. Pollick et al. [49], for example, emphasized that biological motion perception involves substantial observer-level variability related to perceptual experience and social-cognitive functioning.

Within this broader framework, the relationship observed in the present study between RMET performance and valence–arousal judgments derived from unlabeled PLDs may reflect individual differences in sensitivity to subtle socially relevant variations within coordinated movement patterns. This interpretation does not require the assumption that movement contains fixed emotional meanings independent of context or perceiver. Rather, the present findings raise the possibility that socially meaningful affective judgments may emerge through the interaction between structured movement organization and observer-level social–perceptual sensitivity.

This interpretation may also help explain why broad autism-trait measures were less central to the present findings than measures more directly related to social–perceptual interpretation. Studies examining autistic-like trait variation in neurotypical populations should not necessarily be treated as interchangeable with studies involving clinically verified ASD samples, nor should findings derived from one literature automatically be assumed to generalize to the other in the absence of direct empirical evidence. Although these literatures are often discussed together within biological motion research, they involve substantially different participant populations and may not reflect the same underlying perceptual or neurocognitive processes. Consistent with this complexity, studies involving autistic-like traits in neurotypical populations have produced mixed findings, with some reporting altered neural responses or differences in biological motion perception associated with AQ-related variation [54], whereas others have reported more selective effects related to perceptual style or perceptual stability rather than reduced biological motion integration itself [55].

Likewise, studies involving clinically verified ASD samples have also reported heterogeneous findings depending on the specific perceptual and inferential demands of the task. For example, Nackaerts et al. [30] reported reductions in biological motion and emotion recognition in ASD, whereas Saygin et al. [56] found unaffected perceptual thresholds for biological and non-biological form-from-motion perception. Recent work has increasingly emphasized that biological motion perception is not a unitary process, but rather involves multiple partially dissociable components, including perceptual integration of movement structure, interpretation of socially relevant information, and broader observer-level differences in perceptual style or social-cognitive functioning [48,55]. Thus, broad autism-trait variation may not map uniformly onto all forms of movement-based affective inference. Rather, the comparatively stronger relationship observed in the present study between RMET performance and affective judgments derived from unlabeled PLDs may reflect the closer conceptual relationship between the present task and specific forms of social–perceptual interpretation rather than broad autism-trait variation per se.

Although the present findings suggest a relationship between social–perceptual functioning and affective inference from movement, several limitations should be considered when interpreting these results. First, the present study was designed to examine broad relationships between social–perceptual sensitivity and affective judgments derived from unlabeled biological motion rather than isolate the specific movement properties contributing to those judgments. Accordingly, the analyses did not directly identify the precise kinematic or spatiotemporal features underlying participants’ affective interpretations. In addition, the point-light displays were intentionally generated from multiple actors performing relatively natural affective movements rather than highly standardized or prototypical expressions. As a result, individual PLDs likely varied in expressive intensity, ambiguity, movement style, temporal structure, duration, and broader affective organization (“expresser effects”) [57]. This variability was intentionally preserved as part of the relatively natural expressive structure of the stimuli rather than experimentally standardized. Because temporal unfolding itself may contribute to how affective movement is perceived, attempts to standardize movement duration across displays may also impose artificial constraints on the natural spatiotemporal organization of expressive movement. The use of single emotion-specific circumplex reference locations therefore did not capture this stimulus-level variability across individual PLDs. Although this operationalization provided a way to examine how affective meaning may be inferred relative to a theoretically derived circumplex structure, future work may benefit from approaches that model and systematically control stimulus-level variation in affective positioning more directly.

A second limitation concerns the characterization of the participant sample and individual-difference measures. The sample included a greater proportion of female participants than male participants, reflecting the demographics of the available undergraduate participant pool from which recruitment occurred. In addition, demographic variables beyond age and sex were not collected, limiting evaluation of potential demographic influences on affective judgments from biological motion. Prior work has reported sex-related differences in biological motion and bodily emotion recognition tasks involving point-light displays [32], suggesting that sample composition may influence generalizability within this literature. At the same time, Alaerts et al. [32] also reported associations between RMET performance and point-light emotion judgments in a predominantly female sample, indicating that the present findings are not entirely inconsistent with prior work linking social–perceptual sensitivity and biological motion perception. In the present study, however, sex did not significantly contribute to the statistical models, suggesting that the observed relationships were not readily attributable to sex-related variation within the current sample. Nevertheless, future work using more balanced and demographically characterized samples will be important for clarifying the extent to which the present findings generalize across participant populations. In addition, future work would benefit from including broader individual-difference measures, such as sensory processing sensitivity (e.g., [58]) and empathy (e.g., [59]) to clarify whether affective judgments derived from biological motion relate specifically to social–perceptual sensitivity or also reflect broader perceptual and socio-emotional traits.

Finally, the circumplex operationalization itself represents a simplified way of analyzing participants’ valence–arousal judgments. This limitation reflects a broader methodological challenge in the present area: there is not yet a standardized analytic framework for examining how perceivers derive valence–arousal judgments from unlabeled biological motion while preserving natural variability across individual movement displays. In the present study, the reference angles for anger, fear, and happiness were therefore derived from prior circumplex models of affect rather than from an independent norming procedure conducted on the specific PLDs used here. A more stimulus-specific approach would be to obtain independent valence–arousal ratings for each PLD and use the average angular location of each display as its empirical reference point. Those PLD-specific reference locations could then be compared with the valence–arousal judgments of participants in the present task, who were never informed of the actor’s intended affective state. The present study did not include such a norming procedure; therefore, angular-distance values should be interpreted as distances from theoretically derived circumplex reference locations rather than distances from empirically normed affective positions for each individual PLD.

This distinction is important because individual PLDs generated from the same actor-instructed condition likely varied in expressive intensity, ambiguity, movement style, duration, and broader affective quality. As a result, a single reference angle for all anger, fear, or happiness PLDs could not capture stimulus-level differences in how each display might be positioned in valence–arousal space. In addition, valence and arousal are often psychologically interdependent [60], and the circumplex framework itself does not specify which movement properties lead observers to judge a movement as more negative, positive, relaxed, or aroused. Future work would benefit from collecting independent PLD-specific valence–arousal norms and linking those empirically derived affective locations to computational measures of movement kinematics. This would allow researchers to test more directly how particular movement features contribute to affective judgments from human movement.

Despite these limitations, the present study provides evidence that affective judgments can be formed from movement alone and that social–perceptual functioning is systematically related to those judgments. More broadly, the findings support the view that socially meaningful affective judgments may emerge through the interaction between structured movement patterns and perceiver-level sensitivity, even under highly reduced viewing conditions. Establishing these relationships represents an important step toward understanding how movement structure and observer-level perceptual processes jointly contribute to affective inference from human movement.
